# Meiotic long non-coding meiRNA accumulates as a dot at its genetic locus facilitated by Mmi1 and plays as a decoy to lure Mmi1

**DOI:** 10.1098/rsob.140022

**Published:** 2014-06-11

**Authors:** Yuichi Shichino, Akira Yamashita, Masayuki Yamamoto

**Affiliations:** 1 Laboratory of Gene Function, Kazusa DNA Research Institute, 2-6-7 Kazusa-kamatari, Kisarazu, Chiba 292-0818, Japan; 2 Department of Biophysics and Biochemistry, Graduate School of Science, University of Tokyo, Hongo, Tokyo 113-0033, Japan; 3 Laboratory of Cell Responses, National Institute for Basic Biology, Nishigonaka 38, Myodaiji, Okazaki, Aichi 444-8585, Japan

**Keywords:** long non-coding RNA, meiosis, nuclear body, RNA localization, *Schizosaccharomyces pombe*

## Abstract

Long non-coding RNAs (lncRNAs) play key roles in the formation of nuclear bodies. In the fission yeast *Schizosaccharomyces pombe*, a lncRNA species termed meiRNA forms a nuclear dot structure at its own genetic locus, the *sme2* locus, with its protein-binding partner Mei2. This dot structure, called Mei2 dot, promotes the progression of meiosis by suppressing Mmi1, a crucial factor involved in the selective elimination of meiosis-specific transcripts. The meiRNA itself is a target of Mmi1-mediated elimination and is supposed to function as a decoy to lure Mmi1. However, detailed mechanisms underlying the formation of Mei2 dot and inactivation of Mmi1 remain ambiguous. Here, we show that the localization of meiRNA, at its genetic locus *sme2*, depends on its association with Mmi1. We also demonstrate that one of the multiple Mmi1 foci in mitotic cells localizes to the *sme2* locus. Furthermore, the overexpression of meiRNA promotes the accumulation of Mmi1 to the *sme2* locus even in the absence of Mei2 and reduces the activity of Mmi1. These findings indicate that the retention of meiRNA at its genetic locus is facilitated by Mmi1, which then attracts scattered Mmi1 to inhibit its function.

## 2. Introduction

Regulated spatial positioning within the nucleus of eukaryotic cells is important for the achievement of accurate and effective cellular function. To enhance the interaction among components, the nucleus is precisely compartmentalized into subnuclear domains or nuclear bodies [1]. Unlike most cellular organelles, nuclear bodies are non-membrane-bound structures that are thought to be formed by the interaction of components such as proteins and RNAs. Recent studies have revealed that a particular group of long non-coding RNAs (lncRNAs) has a pivotal role in the formation of nuclear bodies [2]. For instance, the lncRNA *NEAT1* is essential for the assembly of the paraspeckle, which is known to retain transcripts in the nucleus of mammalian cells [3–8]. *NEAT1* RNA accumulates at the site of its transcription and serves as a scaffold to recruit paraspeckle proteins, from which paraspeckles spread into the nucleoplasm [3].

In the fission yeast *Schizosaccharomyces pombe*, the Mei2 dot is known as a nuclear body that requires a lncRNA for its formation. The Mei2 dot is composed of Mei2 protein, the master regulator of meiosis in fission yeast, and a lncRNA termed meiRNA [9–11]. The meiRNA, which is encoded by the *sme2* gene, is polyadenylated and has two types of isoforms, meiRNA-S (about 0.5 kb) and meiRNA-L (longer than 1.0 kb) [10,12]. Mei2 physically interacts with meiRNA and forms a dot structure at the *sme2* locus on chromosome II, depending on the transcription of meiRNA [10,11,13]. These observations suggest that the transcribed meiRNA is tethered to its own genetic locus and may play a role as a scaffold for Mei2 dot formation.

The Mei2 dot promotes meiosis by blocking the RNA-binding protein Mmi1, which plays a pivotal role in the selective elimination of meiosis-specific transcripts in vegetatively growing cells [14]. As Mmi1 suppresses the progression of meiosis, it should be inactive when the cells proceed to meiosis. To accomplish this, the Mei2 dot sequesters Mmi1 during the early phase of meiosis, which is observed as scattered nuclear foci in growing cells, so that meiotic transcripts may escape from Mmi1-mediated degradation and achieve stable expression [14].

Target transcripts of Mmi1 harbour a distinct region termed ‘determinant of selective removal’ (DSR), which is enriched with repeats of hexanucleotide UNAAAC motifs [12,15,16]. Mmi1 belongs to the YTH protein family [17] and binds to the DSR region to induce degradation of the transcripts by nuclear exosomes in a polyadenylation-dependent fashion [12,14,15,18,19]. The nuclear exosome component Rrp6 and a zinc finger protein Red1 have been shown to be crucial for the Mmi1/DSR degradation system [14,15,18–21]. Interestingly, recent studies have revealed the participation of Mmi1 and Red1 in facultative heterochromatin formation in a subset of their target genes [16,22,23].

We have reported that meiRNA-L carries numerous copies of the DSR motif in its 3′ region and is indeed a target of Mmi1-mediated degradation, suggesting that meiRNA may act as a decoy for Mmi1 [12]. Furthermore, it has been shown that the meiRNA retained on the chromosome plays a crucial role in the recognition and robust pairing of homologous chromosomes during meiotic prophase [24]. Despite its fundamental importance for the regulation of meiosis, the molecular mechanism underlying the localization of meiRNA to its genetic locus remains ambiguous. In this study, we show that Mmi1 is required to anchor meiRNA to the *sme2* locus and that retained meiRNA has the ability to converge scattered Mmi1 foci into a single dot. We also demonstrate the reduction of the Mmi1 activity by meiRNA. These findings may shed new light on the mechanisms underlying the generation of lncRNA-containing nuclear bodies and meiotic gene expression.

## 3. Results

### 3.1. meiRNA binds to Mmi1 through the 3′ region carrying DSR motifs

**Figure 1.**
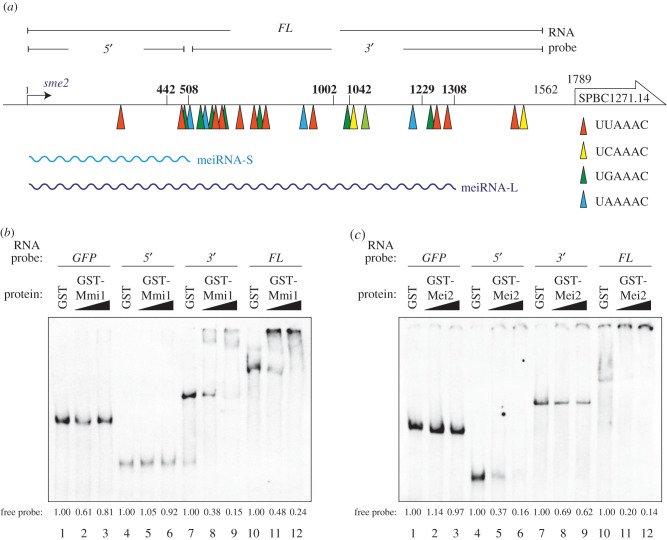
The 3′ region of meiRNA-L carrying DSR motifs binds with Mmi1, and the 5′ region binds with Mei2. (*a*) The location of the hexanucleotide DSR motifs within the *sme2* gene. The UUAAAC, UCAAAC, UGAAAC and UAAAAC sequences are indicated by an orange, a yellow, a green and a blue arrowhead, respectively. Bold numbers indicate 3′-ends of meiRNA. The wavy lines indicate two isoforms of the meiRNA transcript. RNA probes used in the EMSA are shown at the top of the illustration. (*b*) EMSA for interaction between Mmi1 and meiRNA variants. Bacterially expressed and purified GST-Mmi1 (either 20 or 50 nM) or GST portion alone (3 µM) was incubated with DIG-labelled probes of meiRNA variants (0.1 nM) transcribed *in vitro*. The *GFP* transcript was used as a negative control. The relative abundances of free probes are indicated. (*c*) EMSA to detect the interaction between Mei2 and meiRNA variants. Bacterially expressed and purified GST-Mei2 (either 40 or 80 nM) or GST portion alone (3 µM) was incubated with DIG-labelled probes of meiRNA variants (0.2 nM) transcribed *in vitro*. The *GFP* transcript was used as a negative control. The relative abundances of free probes are indicated.

To gain insights into the mechanism underlying meiRNA dot formation, we analysed meiRNA to identify the regions responsible for interaction with Mmi1 or Mei2. We have previously shown that the *sme2* gene carries numerous hexanucleotide motifs that constitute the DSR in the 3′ region, and the meiRNA-L, which is the longer isoform carrying the 3′ tail, may act as a decoy to lure Mmi1 (figure 1*a*) [12]. We performed electrophoretic mobility shift assays (EMSAs) to test whether meiRNA-L is bound to Mmi1 through its 3′ region. Three types of probes were prepared by *in vitro* transcription (figure 1*a*): full-length (*FL*, 1–1562), 5′ portion (*5*′, 1–508) and 3′ portion (*3*′, 509–1562). The full-length and the 5′ portion corresponded to meiRNA-L and meiRNA-S, respectively. Bacterially purified GST-Mmi1, but not control GST, reduced the mobility of the *3*′ (figure 1*b*, lanes 7–9) and *FL* probes (lanes 10–12) to the same extent. It was not effective for the *5*′ probe (lanes 4–6). We previously showed the *in vitro* interaction of a probe corresponding to meiRNA-S with Mmi1 when the amount of Mmi1 is larger [14]. This is consistent with Mmi1 having the ability to recognize *in vitro* a single copy of the DSR motif [12].

The interaction of meiRNA with Mei2 was demonstrated previously by using a probe corresponding to meiRNA-S when the presence of meiRNA-L was not recognized [10]. We reinvestigated the binding between meiRNA and Mei2 using the *FL*, *5*′ and *3*′ probes. Consistent with the previous results, Mei2 could trap the *5*′ (figure 1*c*, lanes 4–6) and *FL* probes (lanes 10–12). However, it failed to form a complex with the *3*′ probe (lanes 7–9). Altogether, these observations indicate that Mmi1 interacts with the 3′ region of meiRNA-L, which carries ample DSR motifs, whereas Mei2 interacts preferentially with the 5′ region of meiRNA-L.

### 3.2. The DSR-enriched 3′ region of meiRNA is responsible for dot formation and the progression of meiosis

**Figure 2.**
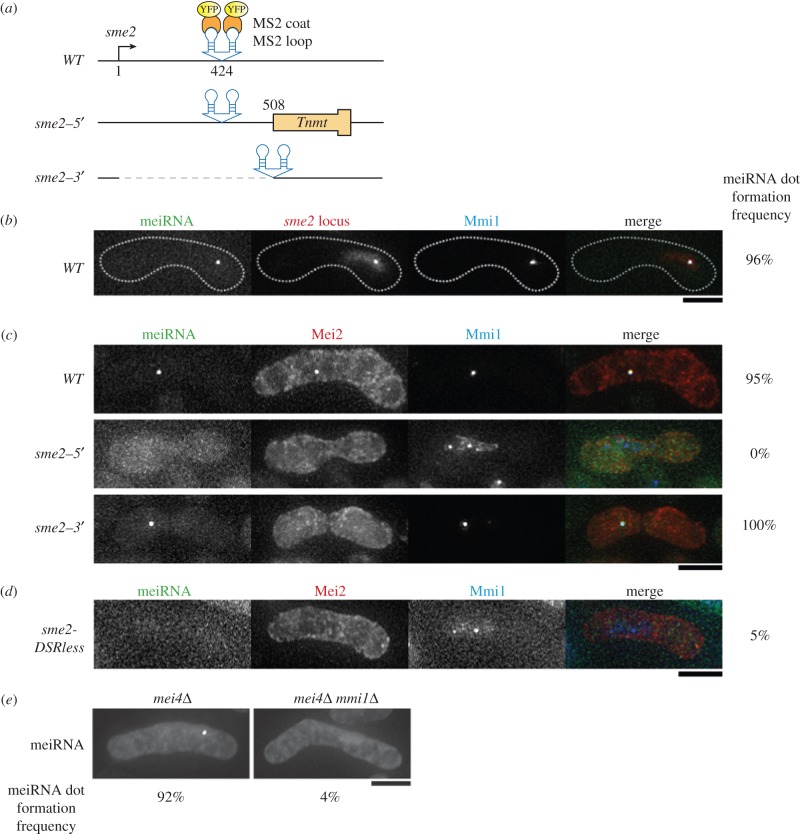
The DSR motifs within meiRNA are required for dot formation. (*a*) Schematic illustration of the meiRNA visualizing system and two *sme2* mutants: *sme2–5*′ and *sme2–3*′. In the *sme2–5*′ mutant, the terminator region of the *nmt1* gene (*Tnmt*) is introduced 508 bp downstream from the transcription start site of *sme2*. In the *sme2–3*′ mutant, *sme2*(*1–508*) is removed. The MS2 loop was inserted in the indicated site. (*b*) Localization of meiRNA with the MS2 system. Wild-type (JS1) cells expressing MS2-loop-tagged meiRNA and CFP-Mmi1 from the respective endogenous promoters, and MS2-YFP and 4mCherry-LacI-NLS from the weakened *adh1* promoter were examined by fluorescence microscopy under meiotic conditions. To mark the *sme2* locus, 32 copies of the *lacO* repeat were inserted about 2 kb upstream of *sme2*. In the merged image, green indicates meiRNA (MS2-YFP), red indicates the *sme2* locus (4mCherry-LacI-NLS) and blue indicates CFP-Mmi1. The dotted lines indicate the shape of the cell. Scale bar, 5 µm. Frequency of meiotic prophase cells containing meiRNA dot is indicated (*n* > 30). (*c*) Localization of meiRNA in the *sme2* mutants. Wild-type (JS2), *sme2–5′* (JS3) and *sme2–3′* (JS4) cells expressing MS2-loop-tagged meiRNA variants, Mei2-mCherry and CFP-Mmi1 from the respective endogenous promoters and MS2-YFP from the weakened *adh1* promoter were examined under meiotic conditions. In the merged images, green indicates meiRNA (MS2-YFP), red indicates Mei2-mCherry and blue indicates CFP-Mmi1. Scale bar, 5 µm; *n* > 30, (*d*) Localization of meiRNA, Mei2 and Mmi1 in *sme2-DSRless* cells. In *sme2-DSRless* mutants, all hexanucleotide DSR motifs were disrupted by point mutations from UNAAAC to UNAAGC. The *sme2-DSRless* cells (JS6) expressing MS2-loop-tagged DSR-less meiRNA, MS2-YFP, Mei2-mCherry and CFP-Mmi1 were examined under meiotic conditions. In the merged image, green indicates meiRNA-DSR-less (MS2-YFP), red indicates Mei2-mCherry and blue indicates CFP-Mmi1. Scale bar, 5 µm; *n* > 30. (*e*) Localization of meiRNA in *mmi1*Δ cells. *mei4*Δ (JS36) and *mmi1*Δ *mei4*Δ (JS37) cells expressing MS2-loop-tagged meiRNA from its endogenous promoter and MS2-GFP from the expression vector pREP81 were examined under meiotic conditions. Scale bar, 5 µm; *n* > 20.

To visualize the subcellular localization of meiRNA in living cells, we used the bacteriophage MS2 system [25]. Two copies of the MS2 stem–loop sequence were introduced in tandem about 400 bp downstream of the transcription start site of meiRNA (figure 2*a*, *WT*). Simultaneous expression of MS2-loop-containing meiRNA and the MS2-YFP or MS2-GFP fusion protein enabled the observation of meiRNA as a single dot in meiotic prophase nuclei, where meiRNA and Mei2 were supposed to form a dot structure and sequester Mmi1 at the *sme2* locus (figure 2*b,c*) [9,11]. We confirmed that the meiRNA dot, visualized by the MS2 system, coincided with the signal of the chromosomal *sme2* locus marked by the LacI-*lacO* system [13] and that of the Mmi1 dot (figure 2*b*). Colocalization of meiRNA-MS2 loop with Mei2 was also confirmed (figure 2*c*, *WT*). These results indicated that the meiRNA dot visualized by the MS2 system indeed reflected the localization of meiRNA.

Using the MS2 system, we examined the localization of several meiRNA variants. We constructed two mutant strains, named *sme2–5′* and *sme2–3′*, which carried either the 5*′* or the 3*′* portion of meiRNA-L, respectively (figure 2*a*). In the *sme2–5′* mutant, the terminator of the *nmt1* gene was inserted 508 bp downstream from the transcription start site of the *sme2* gene in order to exclude the 3*′* region of meiRNA-L from the transcripts. In the *sme2–3′* mutant, the region corresponding to meiRNA-S (residues 1–508) was deleted, so that only the 3*′* region was transcribed. The MS2 loop sequence was introduced to each construct, as illustrated in figure 2*a*. In contrast to wild-type meiRNA, meiRNA lacking the 3*′* region expressed in *sme2–5′* cells did not form a visible dot under the meiotic conditions (figure 2*c*). The Mei2 dot was not detected either, and Mmi1 foci remained scattered around the nucleus in *sme2–5′* cells. This phenotype was similar to that of the *sme2*Δ mutant [11,14].

Meanwhile, meiRNA lacking the 5*′* region formed a dot in *sme2–3′* cells, as observed in the wild-type cells (figure 2*c*). Mmi1 converged into a single dot in the mutant cells, but the Mei2 dot did not form. A similar observation has been reported with meiRNA lacking residues 1–574 [24,26]. These observations suggest that the 3*′* region of meiRNA-L, which binds to Mmi1, has the ability to form a dot structure at its own genetic locus, and that the 5*′* region of meiRNA-L, which can physically interact with Mei2, is essential for Mei2 dot formation.

We next examined expression levels of Mmi1-target genes in *sme2–5′* and *sme2–3′* cells by using northern blot analysis (electronic supplementary material, figure S1*a*). Expression of *mei4* and *ssm4* was detected in *sme2–3′* cells, although lower than that in wild-type cells (lanes 5 and 6). By contrast, expression of these genes was not observed in *sme2–5′* cells (lanes 3 and 4).

Consistently with the expression of Mmi1-target genes, *sme2–3′* cells could sporulate, whereas *sme2–5′* cells could not (electronic supplementary material, figure S1*b*). These observations indicate that meiRNA dot formation and the progression of meiosis are tightly interconnected.

### 3.3. DSR motifs and Mmi1 are crucial for meiRNA dot formation

Based on the importance of the 3′ region of meiRNA for its dot formation, it was anticipated that DSR motifs within meiRNA might be required for the proper localization of meiRNA. We constructed a mutant strain, designated *sme2-DSRless*, in which all the hexanucleotide DSR motifs in *sme2* except the most upstream one were disrupted by substituting TNAAAC with TNAAGC, which has no DSR function [12]. The *sme2-DSRless* allele allowed the accumulation of the long and short forms of meiRNA transcripts even in growing cells (electronic supplementary material, figure S1*a*, lanes 7 and 8), and the *sme2-DSRless* strain was severely impaired in the expression of Mmi1-target genes, *mei4* and *ssm4*, and sporulation (electronic supplementary material, figure S1*a*,*b*), suggesting that the DSR-less meiRNA lost its function to facilitate meiosis. We examined the localization of mutant meiRNA in *sme2-DSRless* cells and found that the DSR-less meiRNA did not reveal specific localization and failed to show the dot structure (figure 2*d*). In these cells, the Mei2 dot was not observed, and Mmi1 foci were scattered in the nucleus, as was seen in *sme2–5*′ mutant cells. Thus, the DSR motifs on the 3′ tail of meiRNA are essential for meiRNA dot formation.

We next investigated the involvement of Mmi1, which physically interacted with DSR motifs, in meiRNA dot formation. Although the disruption of *mmi1* impairs cell growth severely, the growth defect can be alleviated by the deletion of *mei4*, which encodes one of the meiosis-specific key transcription factors [14,27]. Taking advantage of this aspect, we examined the strain with both *mmi1* and *mei4* deletions to evaluate the effect of *mmi1* deletion. Deletion of the *mei4* gene did not affect meiRNA dot formation. In *mmi1*Δ cells, meiRNA failed to form a dot, although meiRNA was highly accumulated (figure 2*e*; electronic supplementary material, figure S2*a*). From these observations, it is suggested that meiRNA dot formation requires the recognition of DSR motifs by Mmi1.

We next examined meiRNA dot formation in mutants in genes related to Mmi1-mediated mRNA elimination or facultative heterochromatin formation (electronic supplementary material, figure S2*b*). In the deletion mutant of the *pab2* genes, which encodes a nuclear poly(A)-binding protein and plays a pivotal role in Mmi1-mediated degradation of meiotic transcripts [15,18,19], most zygotes carried a single meiRNA dot, as in wild-type cells. However, 18% of *pab2*Δ zygotes contained multiple dots, suggesting that Pab2 might have some contribution in meiRNA dot formation. In a temperature-sensitive mutant of the *pla1* gene, which encodes a canonical poly(A) polymerase [18], meiRNA dot was observed. We could also observe the meiRNA dot in the deletion mutant of the *clr4* gene, which encodes a histone methyltransferase essential for heterochromatin formation [28].

### 3.4. DSR motifs on meiRNA attract Mmi1 to the *sme2* locus

**Figure 3.**
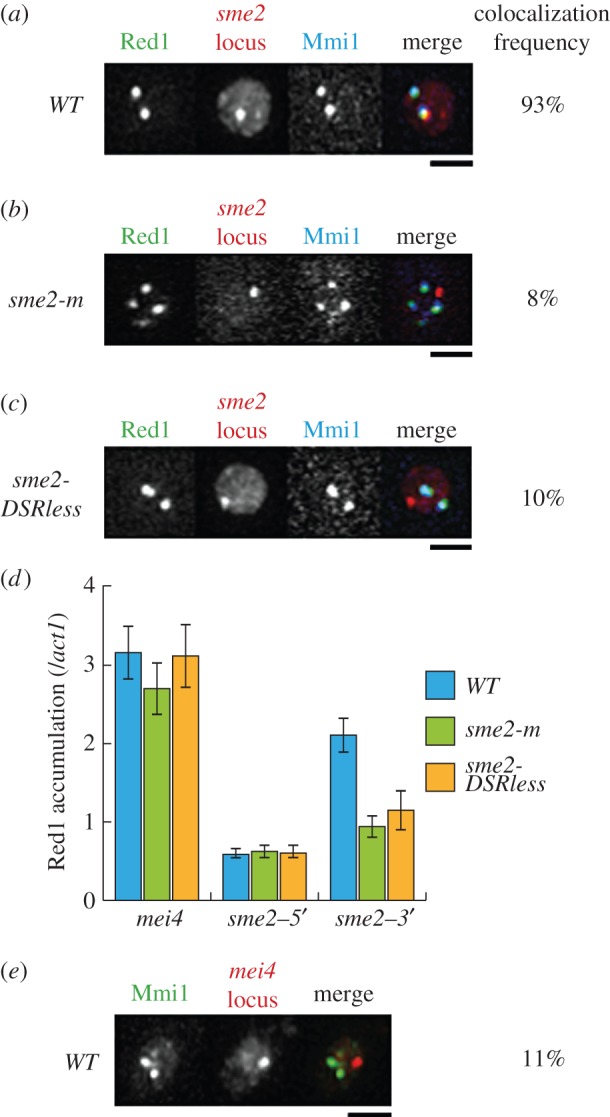
Mmi1 is attracted to the *sme2* locus through the DSR motifs within meiRNA under mitotic conditions. (*a*) Localization of Mmi1 and Red1 in a mitotic nucleus. Wild-type cells (JS8) expressing Red1-YFP and CFP-Mmi1 from the respective endogenous promoters and 4mCherry-LacI-NLS from the weak *adh1* promoter were examined under mitotic conditions. The *lacO* repeats were inserted upstream of *sme2*. Images of the nuclear region are shown. In the merged image, green indicates Red1-YFP, red indicates the *sme2* locus and blue indicates CFP-Mmi1. Scale bar, 2 µm. Frequency of cells in which both Mmi1 and Red1 localized at the *sme2* locus is indicated (*n* > 100). (*b*) Localization of Mmi1 and Red1 in *sme2-m* cells. *sme2-m* cells (JS9) expressing Red1-mCherry, GFP-LacI and CFP-Mmi1 were examined under mitotic conditions. The *lacO* repeats were inserted upstream of *sme2*. Images of the nuclear region are shown. In the merged image, green indicates Red1-mCherry, red indicates the *sme2* locus and blue indicates CFP-Mmi1. Scale bar, 2 µm; *n* > 100. (*c*) Localization of Mmi1 and Red1 in *sme2-DSRless* cells. *sme2-DSRless* (JS10) cells expressing Red1-YFP, 4mCherry-LacI-NLS and CFP-Mmi1 were examined under mitotic conditions. The *lacO* repeats were inserted upstream of *sme2*. Images of the nuclear region are shown. In the merged image, green indicates Red1-YFP, red indicates the *sme2* locus and blue indicates CFP-Mmi1. Scale bar, 2 µm; *n* > 100. (*d*) ChIP analysis of Red1 accumulation on the *mei4* locus and the 5′ or 3′ region of the *sme2* locus in wild-type (JS12), *sme2-m* (JS13) and *sme2-DSRless* (JS14) cells under mitotic conditions, relative to the *act1* locus. Results represent the mean ± s.d. from three reactions. (*e*) Localization of Mmi1 and the *mei4* locus. Wild-type (JS52) cells expressing CFP-Mmi1 and 4mCherry-LacI-NLS were examined under mitotic conditions. The *lacO* repeats were inserted downstream of *mei4*. Images of the nuclear region are shown. In the merged image, green indicates CFP-Mmi1 and red indicates the *mei4* locus. Scale bar, 2 µm; *n* > 100.

In a previous report, we proposed that meiRNA might function as a decoy substrate for Mmi1 to lure it to the *sme2* locus during meiosis [12]. To further investigate the relationship between Mmi1 and the *sme2* genetic locus, we examined Mmi1 foci in mitotically growing cells. In mitotic cells, Mmi1 was observed as one to several scattered nuclear foci [14]. We found that one of the multiple Mmi1 foci formed in mitotic cells was located on the *sme2* locus (figure 3*a*). Red1, which is an essential component for the Mmi1-mediated mRNA elimination [20,21], also showed a similar localization pattern (figure 3*a*). Localization of Mmi1 to the *sme2* locus was not observed in the *sme2-m* mutant, which carries substitution mutations in the TATA-box of the *sme2* promoter and could not transcribe meiRNA [13], indicating that the recruitment of Mmi1 to the *sme2* locus was dependent on the transcription of meiRNA (figure 3*b*). The Mmi1/Red1 foci were also distinct from the *sme2* locus in *sme2-DSRless* mutant cells (figure 3*c*). These observations clearly indicate that meiRNA recruits part of Mmi1 to its own genetic locus even under mitotically growing conditions. We found that the addition of an inhibitor of RNA polymerase II, 1,10-phenanthroline, did not have much impact on the localization of Mmi1 and Red1 on the *sme2* locus (electronic supplementary material, figure S3*a*), suggesting that the transcription of meiRNA might be crucial for the initiation of Mmi1 recruitment.

We next tested the accumulation of Mmi1-mediated elimination machinery in mitotically growing cells by chromatin immunoprecipitation (ChIP) analysis using Red1 as a marker. We detected the accumulation of Red1 at the 3′ region of the *sme2* gene in wild-type cells but not in *sme2-m* or *sme2-DSRless* cells, although Red1 accumulated at the *mei4* gene almost equally in all of them (figure 3*d* and the electronic supplementary material, figure S3*b*).

We further examined whether the other meiotic transcripts could recruit Mmi1 to the genetic locus in mitotic conditions. We found that Mmi1 foci were distinct from the *mei4* locus (figure 3*e*).

### 3.5. meiRNA can converge Mmi1 into a single dot

**Figure 4.**
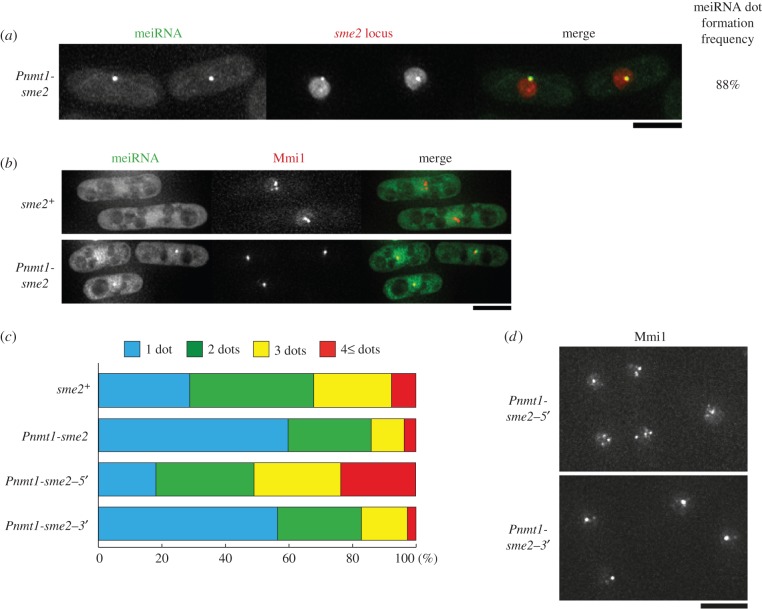
Overexpression of meiRNA causes the convergence of scattered Mmi1 foci. (*a*) Localization of overexpressed meiRNA in mitotically growing cells. Cells (JS16) expressing MS2-loop-tagged meiRNA from the strong *nmt1* promoter, MS2-GFP, and 4mCherry-LacI-NLS from the weakened *adh1* promoter were examined under mitotic conditions. The *lacO* repeats were inserted upstream of *sme2*. In the merged image, green indicates meiRNA (MS2-GFP), and red indicates the *sme2* locus. Scale bar, 5 µm. Frequency of cells containing meiRNA dot is indicated (*n* > 100). (*b*) Localization of mitotic Mmi1 in cells overexpressing meiRNA. *sme2^+^* (JS17) and *Pnmt1-sme2* (JS18) cells expressing MS2-loop-tagged meiRNA, MS2-GFP and CFP-Mmi1 were examined under mitotic conditions. In the merged images, green indicates meiRNA (MS2-GFP), and red indicates CFP-Mmi1. Scale bar, 5 µm. (*c*) Percentages of cells containing 1, 2, 3 or 4 and more Mmi1 dots when *sme2* variants were overexpressed. More than 100 cells were counted in the *sme2^+^* (JS17), *Pnmt1-sme2* (JS18), *Pnmt1-sme2–5′* (JS19) and *Pnmt1-sme2–3′* (JS20) strains under mitotic conditions. (*d*) Localization of mitotic Mmi1 in cells overexpressing *sme2–5′* or *sme2–3′. Pnmt1-sme2–5′* (JS19) and *Pnmt1-sme2–3′* (JS20) cells expressing CFP-Mmi1 were examined under mitotic conditions. Scale bar, 5 µm.

During meiosis, Mmi1 composed a single dot in *sme2–3′* cells, in which Mei2 did not form a dot (figure 2*c*, bottom). This observation suggested that meiRNA itself could converge Mmi1. Therefore, we examined whether the scattered Mmi1 dots in mitotically growing cells could be converged into a single dot by overexpression of meiRNA. Although meiRNA was not detectable in mitotic growing cells that did not overexpress meiRNA (electronic supplementary material, figure S4*a*), it formed a dot at its genetic locus when meiRNA was overexpressed from the strong *nmt1* promoter (*Pnmt1*) (figure 4*a*,*b*), suggesting that the mechanism underlying meiRNA dot formation is functional in mitotic cells. Among these mitotic cells, those that were carrying only a single Mmi1 dot were more frequent (approx. 60%), compared with the frequency of about 30% observed in cells without meiRNA overexpression (figure 4*b*,*c* and the electronic supplementary material, figure S4*b*,*c*). Furthermore, the single Mmi1 dot observed in meiRNA-overexpressing cells was coincident with the dot of overexpressed meiRNA (figure 4*b*). The 3*′* region but not the 5*′* region of meiRNA was responsible for the Mmi1 convergence in mitotic cells (figure 4*c*,*d*).

We next examined whether Mei2 was involved in the ectopic convergence of Mmi1 foci in mitotic cells (electronic supplementary material, figure S5*a*–*e*). Overexpression of the full-length or 3*′* region of meiRNA increased the frequency of mitotic cells carrying a single Mmi1 dot in *mei2*Δ cells as well as in *mei2^+^* cells, indicating that Mei2 is not necessary for this convergence. We further tested whether the overexpression of meiRNA could induce Mei2-independent convergence of Mmi1 foci in meiotic cells. It has been shown that Mmi1 is not converged into a single dot in *mei2*Δ cells under meiotic conditions [14]. When full-length or the 3′ portion of meiRNA was overexpressed in meiotic *mei2*Δ cells, they frequently showed two Mmi1 foci per nucleus (electronic supplementary material, figure S5*f*). As *mei2*Δ cells have been reported to have a defect in telomere clustering, which is a prerequisite for aligning homologous chromosomes before establishment of homologous chromosome paring, and thus show impaired chromosome pairing [29], the two Mmi1 foci might reflect their localization at each *sme2* locus on unpaired homologous chromosomes. These data support the notion that meiRNA itself has the fundamental ability to converge the Mmi1 into one focus.

### 3.6. meiRNA reduces the activity of Mmi1

**Figure 5.**
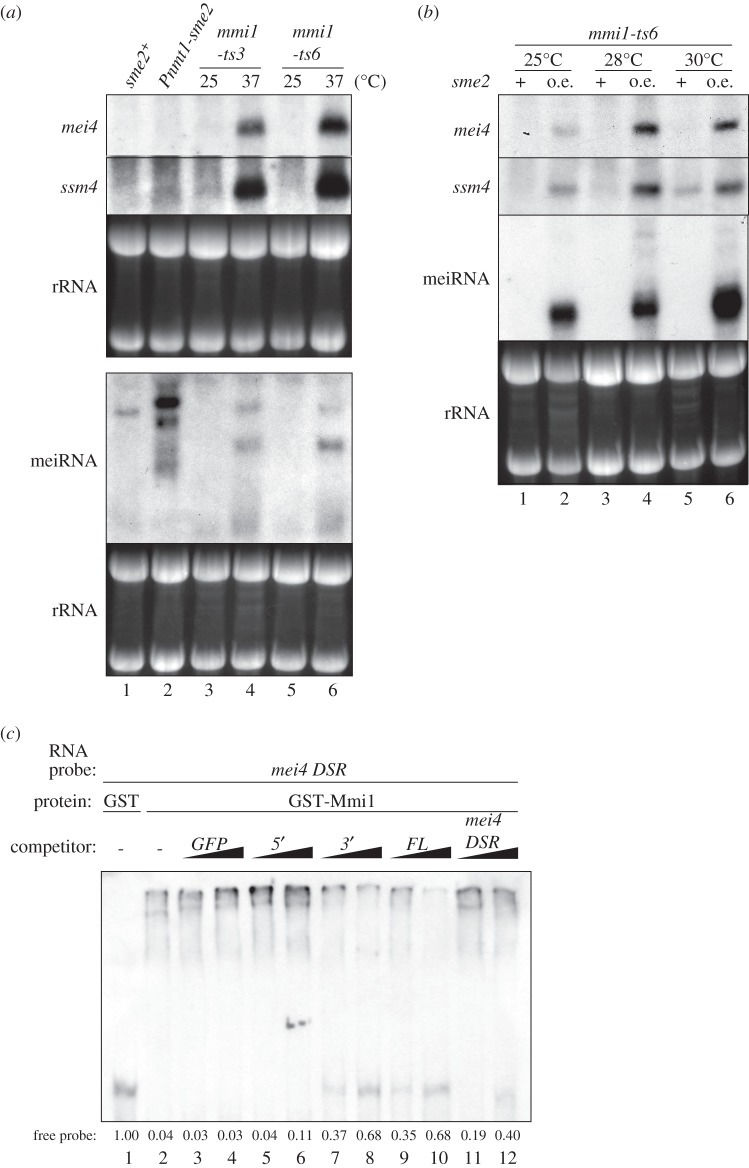
Overexpression of meiRNA reduces the Mmi1 activity. (*a*) Expression of DSR-containing *mei4* and *ssm4* mRNAs was examined by northern blot analysis in *sme2-*overexpressing cells. Wild-type (JS33) and *Pnmt1-sme2* (JS34) cells were grown in MM media at 30°C. *mmi1-ts3* (JV579) and *mmi1-ts6* (JV582) cells were incubated in YE media at 25°C and then shifted to 37°C for 2 h. The rRNAs stained with ethidium bromide are shown in the bottom panel as loading controls. (*b*) Expression of *mei4* and *ssm4* mRNAs was examined by northern blot analysis in *sme2-*overexpressing *mmi1* mutant cells. *mmi1-ts6* (JV582) and *mmi1-ts6 Pnmt1-sme2* (JS35) cells were incubated in MM media at 25°C, 28°C and 30°C, respectively. The rRNAs stained with ethidium bromide are shown as loading controls. (*c*) Sequence-specific competition of meiRNA for the binding between Mmi1 and the DSR region of *mei4* mRNA. GST-Mmi1 (30 nM) or GST portion alone (3 µM) was mixed with DIG-labelled *mei4DSR* transcripts (0.5 nM) and non-labelled competitor meiRNA variants, *mei4DSR* or *GFP* transcripts (5 or 50 nM). The relative abundances of free probes are indicated.

It has been proposed that the Mei2 dot, composed of meiRNA and Mei2, sequesters Mmi1 and suppresses its activity during meiosis [14]. We have shown that meiRNA itself has the ability to converge Mmi1 into a single focus without the aid of Mei2, suggesting that meiRNA can reduce the activity of Mmi1. To test this, we examined whether meiRNA overexpression affects the expression levels of *mei4* and *ssm4* mRNAs, both of which are targets of Mmi1 and are accumulated under mitotically growing conditions when Mmi1 is inactivated by temperature-sensitive mutations (figure 5*a*, lanes 4 and 6). Overexpression of meiRNA had no effect on expression of *mei4* and *ssm4* mRNAs in the wild-type *mmi1* background, although expression levels of meiRNA were comparable in *Pnmt1-sme2* cells and meiotic wild-type cells (figure 5*a*, lane 2, and the electronic supplementary material, figure S6*a*,*b*). However, accumulation of *mei4* and *ssm4* mRNAs was induced by meiRNA overexpression in temperature-sensitive *mmi1* mutant cells at non-restrictive (25°C) and semi-restrictive (28°C and 30°C) temperatures (figure 5*b* and the electronic supplementary material, figure S6*b*). These observations indicate that meiRNA is able to reduce the Mmi1 activity, though it is not solely responsible for complete inactivation of Mmi1. Another possibility might be that overexpression of meiRNA induces transcription of Mmi1-target genes, although no link has been found between meiRNA and transcriptional regulation.

We next performed competitive binding assays to examine whether meiRNA has an impact on the target recognition of Mmi1. Full-length meiRNA and the 3′ region of meiRNA-L could inhibit the binding of Mmi1 to the *mei4DSR* probe, but the 5′ region could not (figure 5*c*). Moreover, the inhibitory effect of full-length and the 3′ region of meiRNA was stronger than that of non-labelled *mei4DSR* RNA. These results suggest that meiRNA competes out the Mmi1 to other DSR mRNAs.

### 3.7. The DSR transcripts form a dot

**Figure 6.**
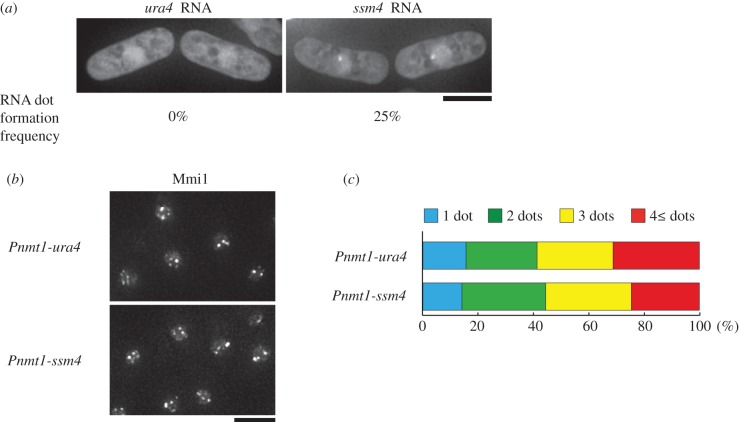
DSR transcripts form a dot. (*a*) Localization of overexpressed *ura4* and *ssm4* RNA in mitotically growing cells. *Pnmt1-MS2loop-ura4* (JS31) and *Pnmt1-MS2loop-ssm4* (JS32) cells expressing MS2-GFP from the expression vector pREP81 were examined under mitotic conditions. Scale bar, 5 µm. Frequencies of cells containing RNA dot are indicated (*n* > 50). (*b*) Localization of mitotic Mmi1 in cells overexpressing *ura4* and *ssm4. Pnmt1-MS2loop-ura4* (JS50) and *Pnmt1-MS2loop-ssm4* (JS51) cells expressing CFP-Mmi1 were examined under mitotic conditions. Scale bar, 5 µm. (*c*) Percentages of cells containing 1, 2, 3 or 4 and more Mmi1 dots when *ura4* or *ssm4* were overexpressed. More than 100 cells were counted in the *Pnmt1-MS2loop-ura4* (JS50) and *Pnmt1-MS2loop-ssm4* (JS51) strains under mitotic conditions.

We finally examined localization of the other DSR-containing transcripts. MS2-loop-tagged *ssm4* RNA could form a single dot when expressed from the strong *nmt1* promoter, although the frequency of the dot formation was not as high as in *sme2*-overexpressing cells (figure 6*a*, 25% versus 88% in figure 4*a*). *ura4* RNA, which carries no DSR region, did not show any specific localization. However, in contrast to meiRNA, overexpressed *ssm4* RNA did not converge Mmi1 into a single dot (figure 6*b*,*c*). These observations suggest that meiRNA has peculiar characteristics among Mmi1-target transcripts.

## 4. Discussion

In this study, we have demonstrated that meiRNA forms a dot at its genetic locus on chromosome II, namely, the *sme2* locus, and that this localization is facilitated by Mmi1. Disruption of either the *mmi1* gene or almost all DSR motifs on meiRNA, which are essential for the binding with Mmi1, impairs meiRNA dot formation. These observations lead us to speculate that meiRNA is recognized by Mmi1 and tethered at its transcriptional site.

It has been shown in the budding yeast *Saccharomyces cerevisiae* that heat-shock mRNAs such as *HSP104* or *SSA4* are retained at or near the respective genetic loci and form dot structures in the presence of a mutation in a canonical poly(A) polymerase *pap1* or mRNA export factors that results in elongation of the 3′ poly(A) tail [30–35]. The retention of these mRNAs depends on the nuclear exosome component Rrp6 [30,32–34]. Interestingly, the canonical poly(A) polymerase (Pla1 in *S. pombe*) and Rrp6 are also key players in the Mmi1-mediated mRNA degradation system [14,15,18,19,21]. We found that cells lacking the nuclear poly(A)-binding protein Pab2 show mild deficiencies in meiRNA dot formation. In 18% *pab2*Δ zygotes, meiRNA forms several dots. This is in clear contrast to *mmi1*Δ, in which meiRNA does not show any specific localization. Pab2 might have some roles in meiRNA dot formation, especially for its maintenance. meiRNA properly forms a dot in the temperature-sensitive *pla1* mutant. However, we cannot rule out the possibility at this stage that Pla1 is involved in meiRNA dot formation, because it might be the case that the residual Pla1 activity in the temperature-sensitive mutant is sufficient to induce or maintain the meiRNA dot. It would be intriguing to determine whether Mmi1-related factors such as Rrp6 are involved in meiRNA dot formation. It has been shown that meiRNA is responsible for the robust pairing of homologues at the *sme2* locus [24]. Mmi1 is likely to participate in the recognition process of homologous chromosomes by regulating localization of meiRNA after telomere clustering, which partially depends on Mei2. It would be interesting to examine the contribution of Mmi1 to the robust pairing.

We previously proposed that meiRNA might function as decoy to lure Mmi1, based on the existence of numerous copies of the DSR motif on it [12]. The following observations obtained in this study reinforce this hypothesis: (i) the DSR-enriched 3′ region of meiRNA binds to Mmi1 directly *in vitro* (figure 1*b*); (ii) the 3′ region has the ability to converge scattered mitotic Mmi1 foci to the *sme2* locus (figure 4), while *ssm4* RNA does not (figure 6*b*,*c*); and (iii) the 3′ region hampers the binding of Mmi1 to another DSR region (figure 5*c*). Furthermore, we have found that one of the Mmi1 foci localizes to the *sme2* locus even in mitotically growing cells (figure 3*a*,*d*). On the other hand, Mmi1 foci do not coincide with the *mei4* locus (figure 3*e*). Because the expression of meiRNA is boosted when the cells enter the meiotic cell cycle [10], this might lead to the full recruitment of Mmi1 to the *sme2* locus, as has been shown with the overexpression of meiRNA in mitotic cells.

It may be noteworthy, however, that overexpression of meiRNA does not lead to such severe growth retardation as does inactivation of Mmi1. Furthermore, the reduction of Mmi1 activity by the overexpression of meiRNA can be observed only when Mmi1 is partially inactivated (figure 5*b*). These observations suggest that sequestration of Mmi1 to the *sme2* locus alone may not be sufficient to inactivate Mmi1. Complete inactivation of Mmi1 might be accomplished by the function of Mei2, which also forms a complex with meiRNA at the *sme2* locus [11,13]. In addition to enhanced meiRNA expression, the production and activation of Mei2 are enhanced when the cells enter the meiotic cell cycle [9,36,37]. Hence, it is presumable that Mei2 dampens the activity of Mmi1 lured to the *sme2* locus. In support of this notion, Mei2 and Mmi1 can physically interact by themselves [14], though it remains unclear how Mei2 dampens the function of Mmi1. We have demonstrated that Mei2 is dispensable for the convergence of Mmi1 foci by the overexpression of meiRNA (electronic supplementary material, figure S5). Mmi1 does not converge into a single spot in *mei2*Δ cells without meiRNA overexpression, in which meiRNA does not form a nuclear dot [11,14]. Mei2 might induce expression of meiRNA as with other DSR transcripts by inhibiting the Mmi1 activity, and cause the further inactivation of Mmi1 in cooperation with meiRNA. We previously showed that Mei2 forms a dot in *sme2*Δ cells when *ssm4* is overexpressed from a high-copy number plasmid [11]. We have found that *ssm4* RNA overexpressed from the strong promoter on a chromosome does not recruit Mmi1 to its locus in mitotically growing cells. We assume that the dot of Mei2 observed in *sme2*Δ cells overexpressing *ssm4* is distinct from the site of the *ssm4* gene in the high-copy number plasmid, or the *ssm4* locus, because Mei2 does not directly interact with DSR RNAs. It might be noted that the *sme2*Δ strain used in these experiments carries the 3′ region of meiRNA-L, although the 3′ region and the promoter region are split by an auxotrophic marker cassette [10,11]. Further experiments are required to determine where Mei2 localizes in this case.

It is not clear how meiRNA is tethered at its own genetic locus. *Xist* RNA, a pivotal factor for X chromosome inactivation (XCI) in mammalian female cells, is one of the best-characterized lncRNAs that functions via interaction with a chromosome [38]. *Xist* RNA is expressed only from the future inactive X chromosome (Xi) and is initially localized at a chromosomal region essential for XCI, known as the X-inactivation centre (*Xic*), within the *Xist* gene [39–43]. Tethering of *Xist* RNA to *Xic* requires a ‘bivalent’ protein YY1, capable of binding both *Xist* RNA and *Xic* at the Xi allele [44]. Protein(s) similar to YY1 might possibly be involved in the tethering of meiRNA by bridging meiRNA (or Mmi1) and the *sme2* locus.

Another interesting and unsolved issue is where in the nucleus DSR-carrying transcripts are degraded. Although the exact localization of multiple mitotic Mmi1 foci has not been elucidated, we have demonstrated that one of them localizes to the *sme2* locus. The number of Mmi1 foci detected in mitotically growing cells ranges from one to several [14], and if cells carry only a single Mmi1 focus, it localizes to the *sme2* locus, raising the possibility that the *sme2* locus is the major site of degradation. Identification of the site for Mmi1-mediated mRNA degradation may provide new perspective to the study of nuclear bodies and RNA metabolism.

## 5. Material and methods

### 5.1. Yeast strains, genetic methods and growth media

The *S. pombe* strains used in this study are listed in the electronic supplementary material, table S1. The general genetic methods used to analyse the *S. pombe* strains have been previously described [45]. Standard gene-targeting protocols were used to create deletion mutants and epitope-tagged strains [46,47]. Growth medium included complete medium YE, minimal medium SD and MM [48], synthetic sporulation medium SSA [49] and sporulation agar SPA [45].

To visualize meiRNA in living cells, we used the MS2 system [25]. Two copies of an MS2 loop sequence that is specifically recognized by an MS2 bacteriophage coat protein were introduced within the *sme2* gene. The MS2 coat protein was fused with fluorescence proteins.

The *CO2* region, which is the gene-free region between SPAC2E1P3.05c and SPAPB2C8.01 on chromosome I, was selected as a target of chromosomal integration of cloned constructs.

Labelling of chromosomal loci near the *sme2* and *mei4* gene by using a *lac* repressor (*lacI*)/*lac* operator (*lacO*) recognition system was carried out as previously described [13,50,51]. Thirty-two copies of a *lacO* repeat were integrated into the approximately 2 kb upstream region of the *sme2* gene (between *mrpl8* and *kes1*) or the approximately 0.5 kb downstream region of the *mei4* gene (between *mei4* and *act1*). The mCherry-tagged LacI was expressed from the *adh41* promoter, which carried mutations in the TATA sequence of the *adh1* promoter (TATAAATA to ATAAA). Primers used for *lacO* integration are listed in the electronic supplementary material, table S2.

Truncation mutations of the *sme2* gene were constructed by using the PrimeSTAR Mutagenesis Basal Kit (Takara). The *sme2-DSRless* gene was constructed by using a DNA fragment synthesized by the gene synthesis service (Operon).

### 5.2. Fluorescence microscopy

Live-cell imaging was performed with the DeltaVision-SoftWoRx system (GE Healthcare). Cells growing in the logarithmic phase in YE or MM liquid medium at 30°C or undergoing mating and meiosis on SPA medium for 4–6 h at 30°C were mounted onto glass bottom culture dishes (MatTek) precoated with lectin, and the dishes were filled with liquid medium. Images were acquired as serial sections along the *z*-axis at 0.5 µm intervals, deconvolved and stacked using the ‘quick projection’ algorithm in the SoftWoRx software.

### 5.3. Northern blot

Northern blot analysis was performed as previously described [11] using DNA probes for the specified genes. Ten micrograms of total cellular RNA was used for each sample.

### 5.4. Electrophoretic mobility shift assays

An RNA EMSA was performed as previously described [12]. Samples were electrophoresed by polyacrylamide gel for 2 h at 110 V and transferred to the GeneScreen Plus membrane (NEN) for 2 h at 200 mA using 0.5× tris–borate–EDTA buffer. The concentrations of DIG-labelled or non-labelled RNAs and bacterially purified proteins are indicated in the figure legends. In competitive binding assays, we used a *mei4DSR* probe, which corresponds to 486–828 bp of the *mei4* gene.

### 5.5. Chromatin immunoprecipitation

Cells were grown to 6.0–8.0 × 10^6^ cells ml^−1^ and cross-linked with 1% formaldehyde at 30°C for 10 min. The reaction was stopped with glycine at a final concentration of 125 mM for 5 min. Cells were collected and washed twice with buffer 1 (50 mM HEPES (pH 7.5), 140 mM NaCl, 1 mM EDTA (pH 7.5), 1% Triton-X, 0.1% sodium deoxycholate) and frozen at −80°C. Cell pellets were suspended in 200 µl of lysis buffer (buffer 1 with protease inhibitor cocktail (Complete Mini EDTA-free; Roche) and phenylmethylsulfonyl fluoride at a final concentration of 1 mM) and lysed using glass beads. Lysates were sonicated (Branson Sonifier 450) and centrifuged. Ten microlitres of each supernatant was saved as whole-cell extracts, and the rest was subject to immunoprecipitation.

For immunoprecipitation, anti-Myc (9E10; Santa Cruz Biotechnology) and anti-HA (negative control, 16E12; Covance) antibodies were pre-bound to magnetic beads (Dynabeads protein G; Life Technologies). Cell extracts were incubated with antibody-coupled beads at 4°C overnight. Beads were washed once with buffer 1, once with buffer 1′ (50 mM HEPES (pH 7.5), 500 mM NaCl, 1 mM EDTA (pH 7.5), 1% Triton-X, 0.1% sodium deoxycholate), once with buffer 2 (10 mM Tris-HCl (pH 8.0), 250 mM LiCl, 5% NP-40, 0.5% sodium deoxycholate) and once with TE (10 mM Tris-HCl (pH 8.0), 1 mM EDTA (pH 8.0)). To de-cross-link, the whole-cell extracts and beads were suspended with 100 µl of 1% SDS in TE and incubated at 65°C overnight. After delinking, the samples were incubated with 1 µl RNaseA (1 mg ml^−1^) at 37°C for an hour, and 1 µl proteinase K (20 mg ml^−1^) at 37°C for 3 h. DNA was extracted twice with phenol : chroloform : isoamyl alcohol and purified by the MinElute PCR Purification Kit (Qiagen). DNA in whole-cell extracts or immunoprecipitates was amplified and analysed by quantitative PCR. Quantitative PCR was carried out using SYBR Premix Ex Taq II (Tli RNaseH Plus, Takara) on the 7300 Real-Time PCR system (Applied Biosystems). The *act1* gene encoding actin was used for normalization. Primers used for quantitative PCR are listed in the electronic supplementary material, table S2.

## Supplementary Material

Supplementary
