# Supplementary material for: Meiotic long non-coding meiRNA accumulates as a dot at its genetic locus facilitated by Mmi1 and plays as a decoy to lure Mmi1
Source: Open Biol. 2014 Jun 11;4(6):140022. doi: 10.1098/rsob.140022 (PMC4077057; doi:10.1098/rsob.140022)
Supplement: Supplementary [file rsob-14-0022-File003.pdf]

Figure S1

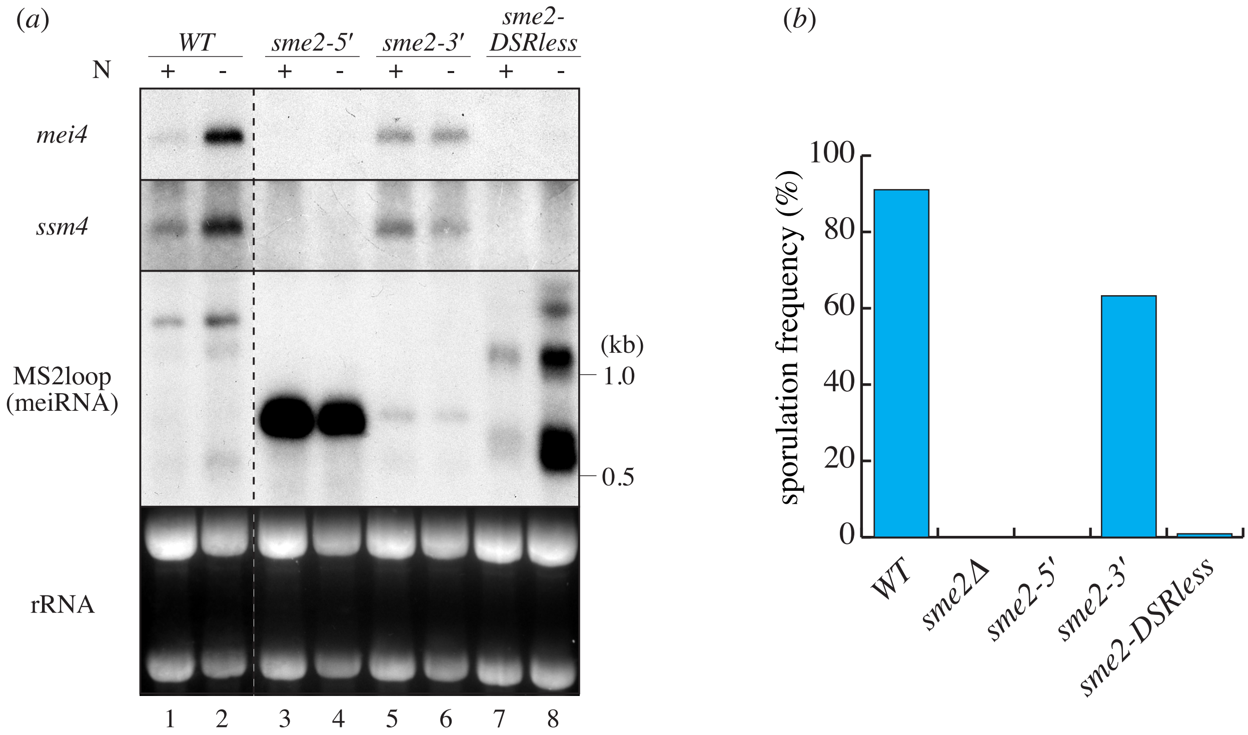

Figure S1. The DSR motifs in meiRNA are crucial for its function.
(*a*) Expression of the meiotic transcripts was examined by northern blot analysis in *sme2* mutants. Wild-type (JS41), *sme2-5′* (JS42), *sme2-3′* (JS43) and *sme2-DSRless* (JS44) cells were grown in MM media at 30°C (N+) and then transferred to MM media without nitrogen source for four hours at 30°C (N-). The rRNAs stained with ethidium bromide are shown in the bottom panel as loading controls. (*b*) Sporulation frequencies of *sme2* mutants. Wild-type (JS38), *sme2Δ* (JT926), *sme2-5′* (JS39), *sme2-3′* (JS40) and *sme2-DSRless* (JS5) cells was measured after incubation on an SPA medium at 30°C for 24 hours ($n > 200$).

Figure S2

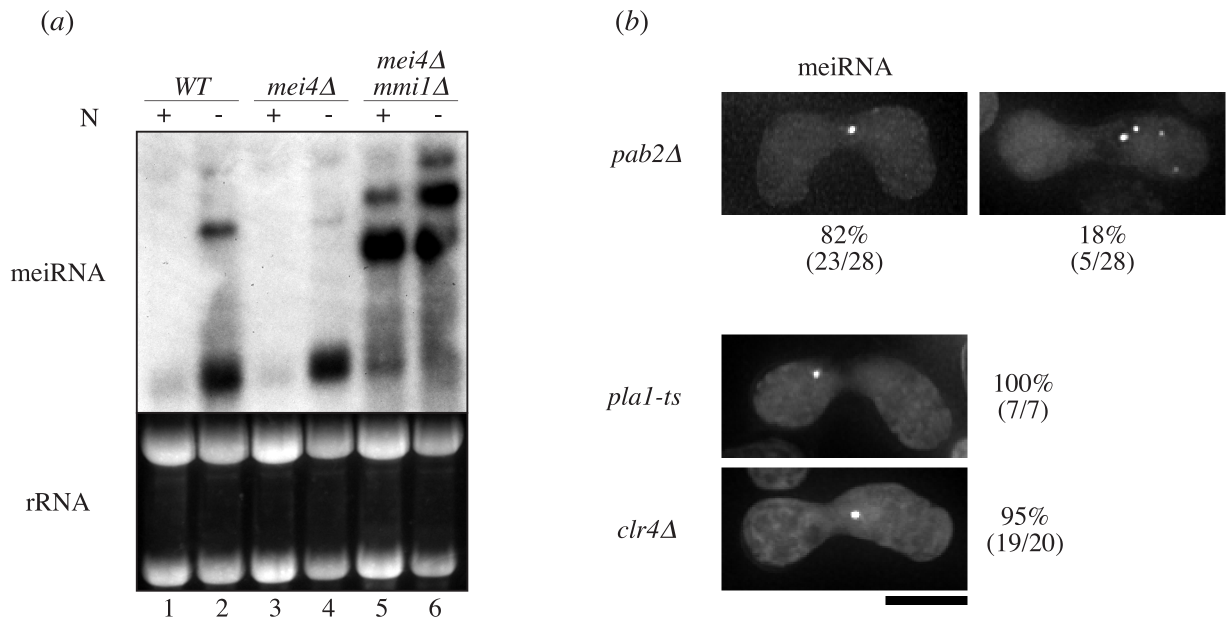

Figure S2. Localization of meiRNA in Mmi1-related mutants.

(*a*) Expression of meiRNA was examined by northern blot analysis in *mmi1*-depleted cells. Wild-type (JY362), *mei4Δ* (JZ807) and *mmi1Δ mei4Δ* (JS45) cells were grown in MM media at 30°C (N+) and then transferred to MM media without nitrogen source for four hours at 30°C (N-). The rRNAs stained with ethidium bromide are shown in the bottom panel as loading controls. (*b*) Localization of meiRNA in mutants involved in Mmi1-mediated meiotic mRNA elimination and facultative heterochromatin formation. *pab2Δ* (JS46), *pla1-ts* (JS47) and *clr4Δ* (JS48) cells expressing MS2 loop-tagged meiRNA from its endogenous promoter and MS2-GFP from the expression vector pREP81 were examined under meiotic conditions. Frequencies of meiotic prophase cells containing meiRNA dot are indicated. Scale bar, 5 μm.

Figure S3

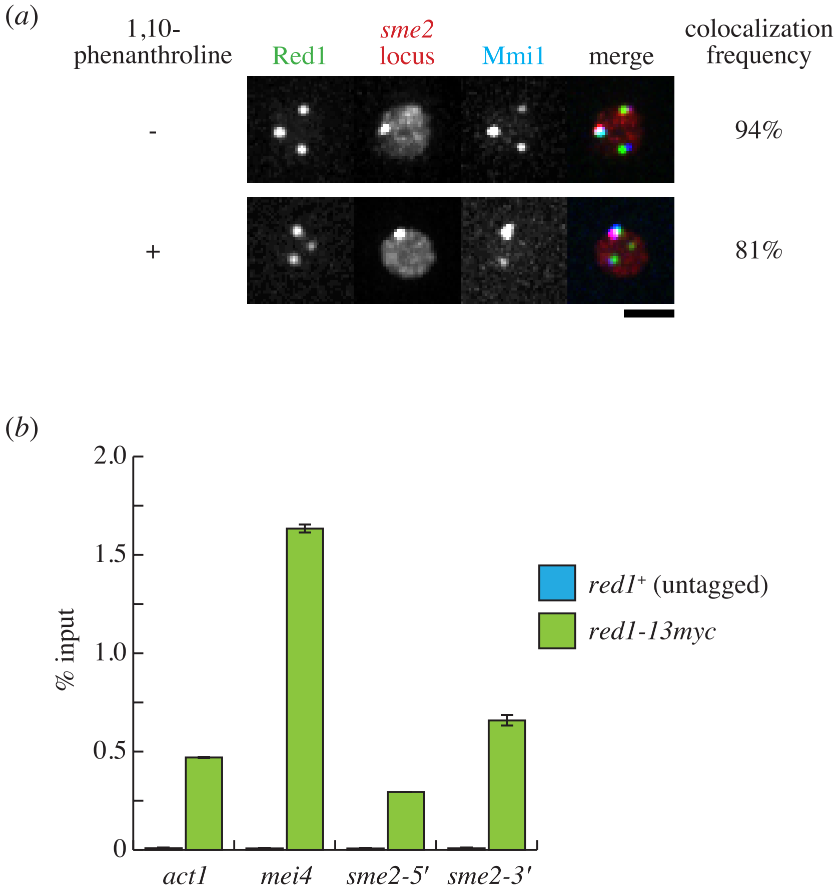

Figure S3. Localization of Mmi1 and Red1 at the *sme2* locus.
(*a*) Localization of Mmi1 and Red1 when transcription is inhibited. Wild-type cells (JS8) expressing Red1-YFP and CFP-Mmi1 from the respective endogenous promoters were grown in YE media and then added with 1,10-phenanthroline (250 μg/mL at final concentration) to inhibit transcription. The LacI-*lacO* system were used to visualize the *sme2* locus. Images of the nuclear region after one-hour incubation with or without 1,10-phenanthroline are shown. In the merged image, green indicates Red1-YFP, red indicates the *sme2* locus, and blue indicates CFP-Mmi1. Scale bar, 2 μm. Frequencies of cells in which Mmi1 and Red1 localized to the *sme2* locus are indicated ($n > 100$). (*b*) Chromatin immunoprecipitation (ChIP) analysis of Red1 accumulation on the *act1* locus, the *mei4* locus and the 5′ or 3′ region of the *sme2* locus in untagged (JY450) and Myc-tagged (JS12) *red1*$^+$ cells under mitotic conditions. Results represent the mean ± standard deviation from three reactions.

Figure S4

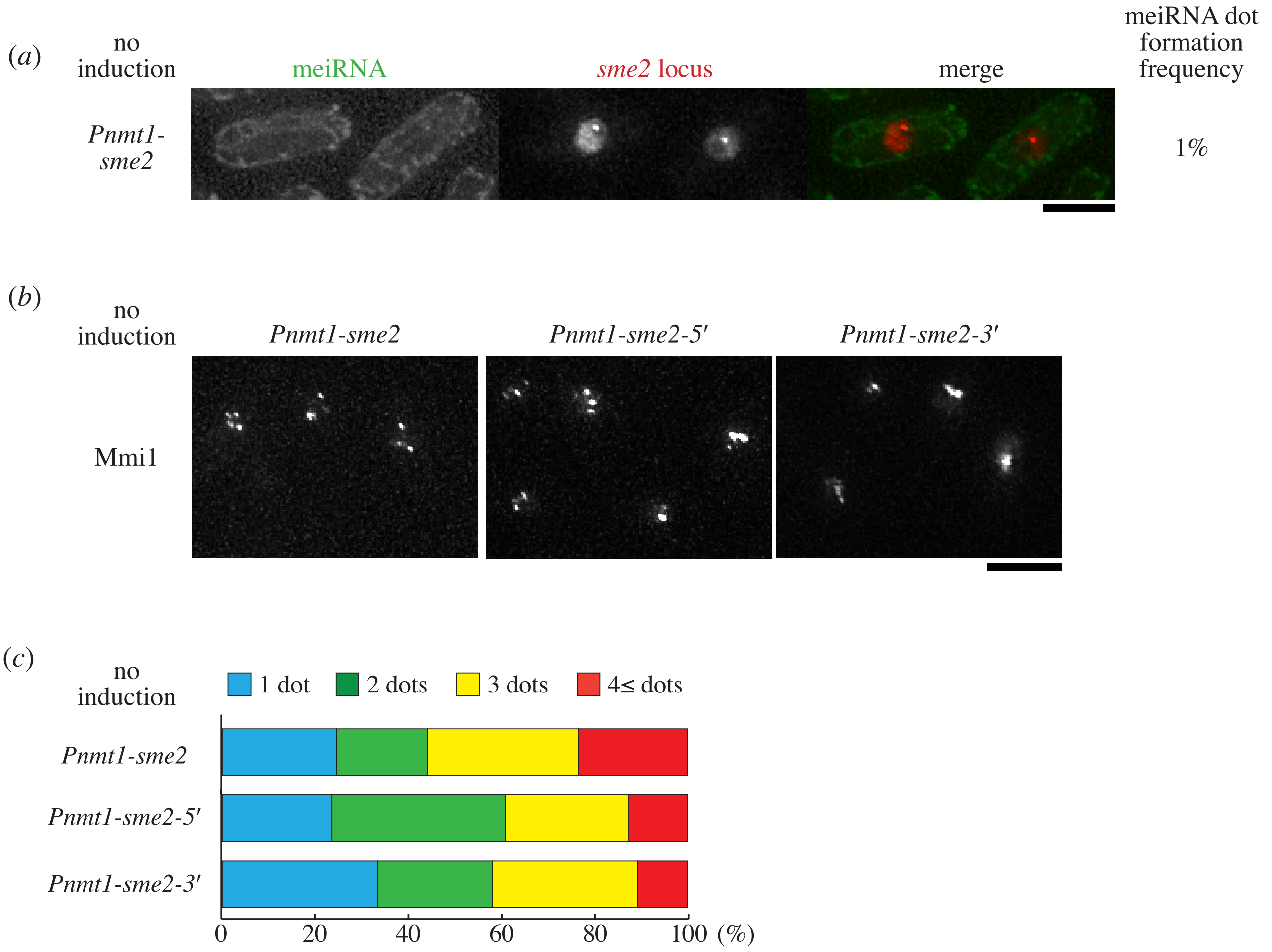

Figure S4. meiRNA does not form a dot in mitotic cells without *sme2* overexpression.
(*a*) Localization of meiRNA in mitotic cells in which *sme2* overexpression was not induced. *Pnmt1-sme2* cells (JS16) were incubated in YE medium. The LacI-*lacO* system were used to visualize the *sme2* locus. In the merged image, green indicates meiRNA (MS2-GFP), and red indicates the *sme2* locus. Scale bar, 5 μm. Frequency of cells containing a meiRNA dot is indicated ($n > 100$). (*b*) Localization of mitotic Mmi1 in cells in which overexpression of *sme2* variants were not induced. *Pnmt1-sme2* (JS18), *Pnmt1-sme2-5′* (JS19) and *Pnmt1-sme2-3′* (JS20) cells expressing CFP-Mmi1 were incubated in YE media. Scale bar, 5 μm. (*c*) Percentages of cells containing 1, 2, 3, or 4 and more Mmi1 dots when overexpression of *sme2* variants were not induced. More than 100 cells were counted in *Pnmt1-sme2* (JS18), *Pnmt1-sme2-5′* (JS19) and *Pnmt1-sme2-3′* (JS20) strains incubated in YE media.

Figure S5

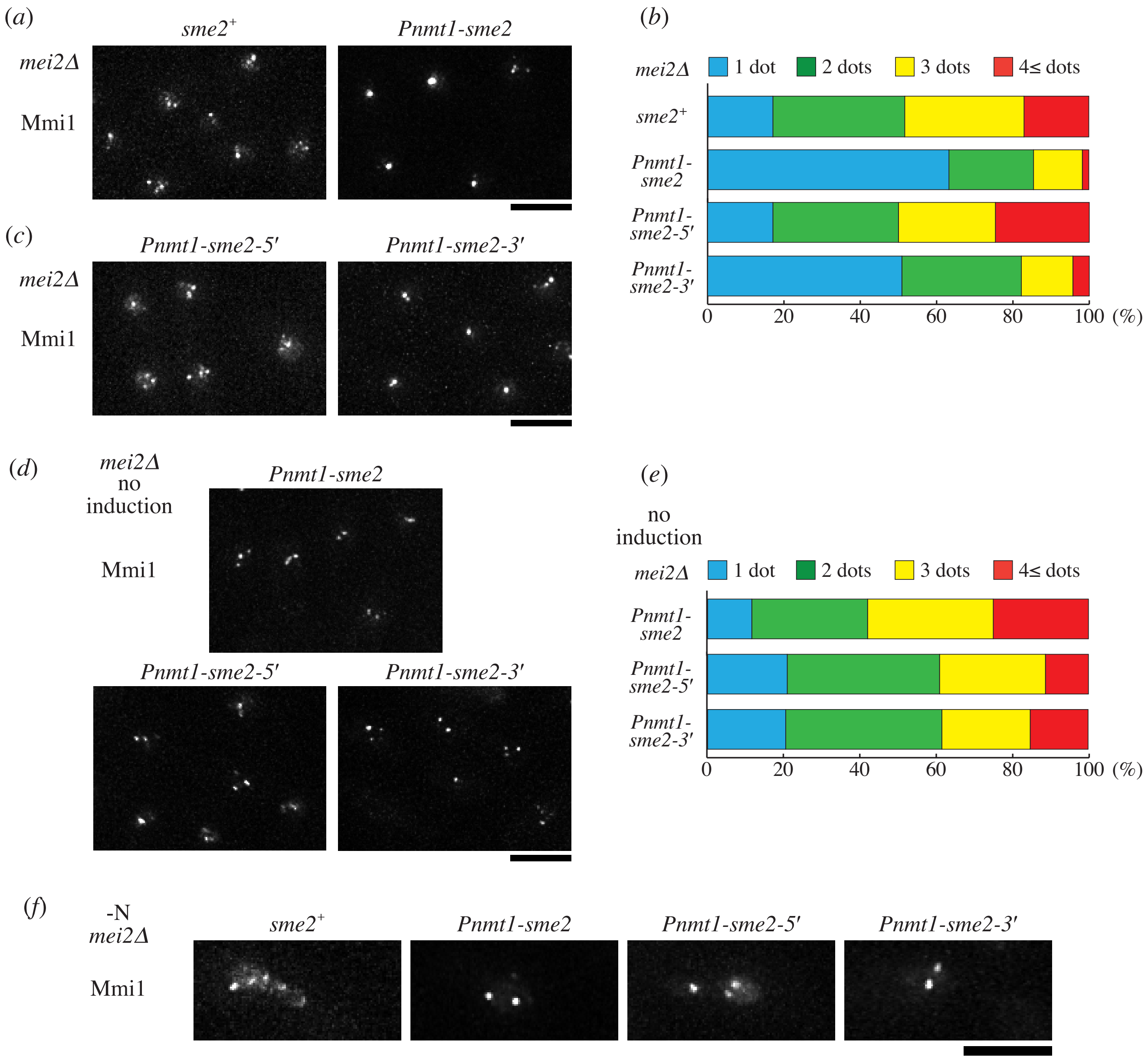

Figure S5. Mei2 is not involved in the convergence of Mmi1 dots by the overexpression of meiRNA.
(*a*) Localization of mitotic Mmi1 in *mei2Δ* cells overexpressing meiRNA. *sme2*$^{+}$ (JS21) and *Pnmt1-sme2* (JS22) cells expressing CFP-Mmi1 with the deletion of *mei2* were examined under mitotic conditions. Scale bar, 5 μm. (*b*) Percentages of cells containing 1, 2, 3, or 4 and more Mmi1 dots when *sme2* variants were overexpressed in *mei2Δ* cells. More than 100 cells were counted in the *sme2*$^{+}$ (JS21), *Pnmt1-sme2* (JS22), *Pnmt1-sme2-5′* (JS23), and *Pnmt1-sme2-3′* (JS24) strains with the deletion of *mei2* under mitotic conditions. (*c*) Localization of mitotic Mmi1 in *mei2Δ* cells overexpressing *sme2-5′* or *sme2-3′*. *Pnmt1-sme2-5′* (JS23) and *Pnmt1-sme2-3′* (JS24) cells expressing CFP-Mmi1 with the deletion of *mei2* were examined under mitotic conditions. Scale bar, 5 μm. (*d*) Localization of mitotic Mmi1 in *mei2Δ* cells in which overexpression of *sme2* variant was not induced. *Pnmt1-sme2* (JS22), *Pnmt1-sme2-5′* (JS23) and *Pnmt1-sme2-3′* (JS24) cells expressing CFP-Mmi1 with the deletion of *mei2* were incubated in YE media. Scale bar, 5 μm. (*e*) Percentages of cells containing 1, 2, 3, or 4 and more Mmi1 dots when overexpression of *sme2* variants were not induced. More than 100 cells were counted in *Pnmt1-sme2* (JS22), *Pnmt1-sme2-5′* (JS23) and *Pnmt1-sme2-3′* (JS24) strains incubated in YE media. (*f*) Localization of meiotic Mmi1 in *mei2Δ* cells overexpressing *sme2* variants. *sme2*$^{+}$ (JS21), *Pnmt1-sme2* (JS22), *Pnmt1-sme2-5′* (JS23), and *Pnmt1-sme2-3′* (JS24) cells expressing CFP-Mmi1 with the deletion of *mei2* were examined under meiotic conditions. Scale bar, 5 μm.

Figure S6

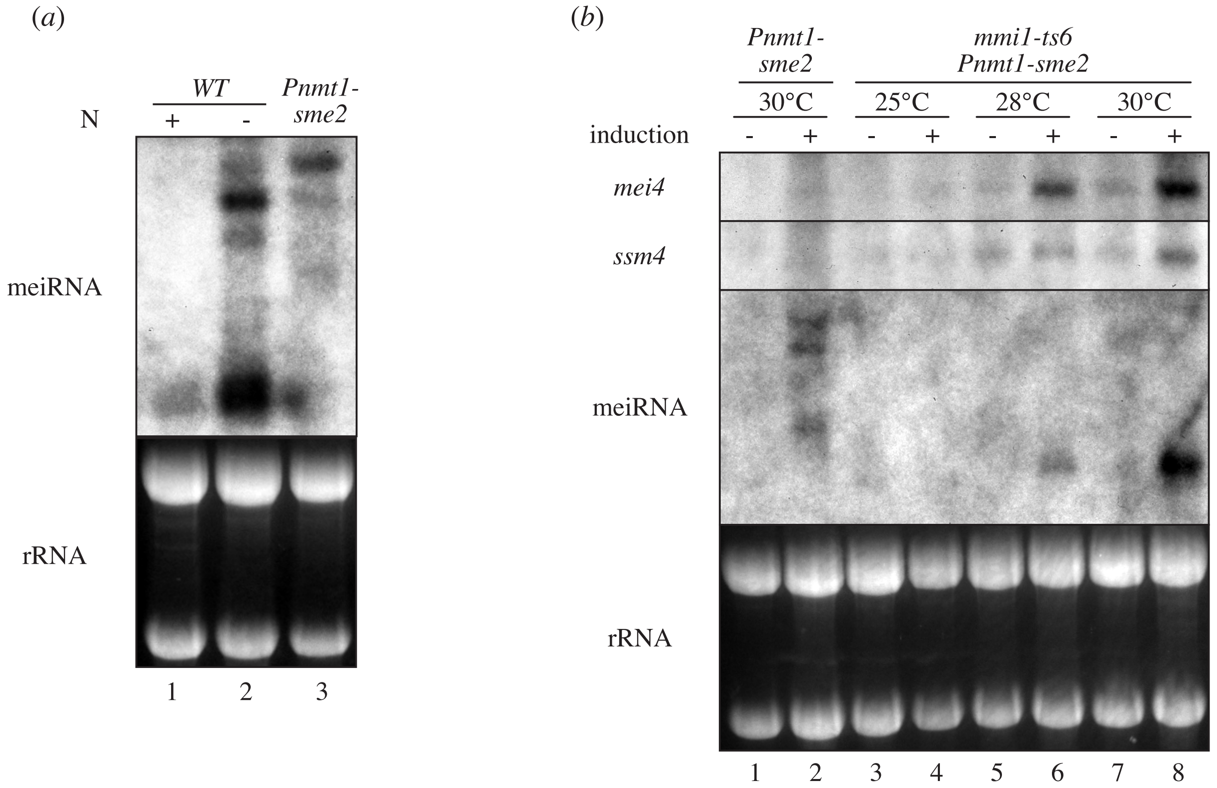

Figure S6. The overexpression of meiRNA reduces the activity of Mmi1.

(*a*) Expressions of meiRNA in meiotic wild-type cells and mitotic *sme2*-overexpressing cells were examined by northern blot analysis. Wild-type (JY362) cells were grown in MM media at 30°C (N+) and then transferred to MM media without nitrogen source for four hours at 30°C (N-). *Pnmt1-sme2* (JS34) cells were grown in MM media. The rRNAs stained with ethidium bromide are shown in the bottom panel as loading controls. Note that overexpressed meiRNA carries the MS2 loop sequence and is about 130 nt longer than wild-type meiRNA. (*b*) Expression of *mei4* and *ssm4* mRNAs was examined by northern blot analysis in *Pnmt1-sme2* (JS34) and *mmi1-ts Pnmt1-sme2* (JS35) cells with or without meiRNA overexpression. These cells were grown in YE media (induction-) and MM media (induction+) at indicated temperatures. The rRNAs stained with ethidium bromide are shown in the bottom panel as loading controls.

Table S1. *S. pombe* strains used in this study

Name	Genotype
JS1	h^{90} lacOx32-kan^{R}-ura4^{+}-sme2-MS2loop-2 arg1::Padh41-4mCherry-lacI-NLS-hph^{R} CO2::Padh41-MS2-YFP-nat^{R} LEU2-CFP-mmi1 ade6-M216 leu1 ura4-D18
JS2	h^{90} bsd^{R}-sme2-MS2loop-2 CO2::Padh41-MS2-YFP-nat^{R} LEU2-CFP-mmi1 mei2-mCherry-hph^{R} ade6-M216 leu1 ura4-D18
JS3	h^{90} sme2-5′-MS2loop-2-Tnmt CO2::Padh41-MS2-YFP-nat^{R} LEU2-CFP-mmi1 mei2-mCherry-hph^{R} ade6-M216 leu1 ura4-D18
JS4	h^{90} MS2loop-2-sme2-3′ CO2::Padh41-MS2-YFP-nat^{R} LEU2-CFP-mmi1 mei2-mCherry-hph^{R} ade6-M216 leu1 ura4-D18
JS5	h^{90} bsd^{R}-sme2-DSRless-MS2loop-2 ade6-M216 leu1 ura4-D18
JS6	h^{90} bsd^{R}-sme2-DSRless-MS2loop-2 CO2::Padh41-MS2-YFP-nat^{R} LEU2-CFP-mmi1 mei2-mCherry-hph^{R} ade6-M216 leu1 ura4-D18
JS8	h^{90} lacOx32-kan^{R}-ura4^{+}-sme2-MS2loop-2 arg1::Padh41-4mCherry-lacI-NLS-hph^{R} LEU2-CFP-mmi1 red1-YFP-nat^{R} ade6-M216 leu1 ura4-D18
JS9	h^{90} lacOx32-kan^{R}-ura4^{+}-LEU2-sme2-m nat^{R}-CFP-mmi1 red1-mCherry-hph^{R} his7^{+}<<GFP-lacI ade6-M210 leu1 ura4-D18
JS10	h^{90} lacOx32-kan^{R}-ura4^{+}-sme2-DSRless-MS2loop-2 arg1::Padh41-4mCherry-lacI-NLS-hph^{R} LEU2-CFP-mmi1 red1-YFP-nat^{R} ade6-M216 leu1 ura4-D18
JS12	h^{90} red1-13myc-nat^{R} ade6-M216 leu1
JS13	h^{90} lacOx32-kan^{R}-ura4^{+}-LEU2-sme2-m red1-13myc-nat^{R} ade6-M216 leu1 ura4-D18
JS14	h^{90} bsd^{R}-sme2-DSRless-MS2loop-2 red1-13myc-nat^{R} ade6-M216 leu1
JS16	h^{90} lacOx32-kan^{R}-ura4^{+}-bsd^{R}-Pnmt1-sme2-MS2loop-2 arg1::Padh41-4mCherry-lacI-NLS-hph^{R} CO2::Padh81-MS2-GFP-nat^{R} ade6-M216 leu1 ura4-D18
JS17	h^{90} sme2-MS2loop-2-bsd^{R} nat^{R}-CFP-mmi1 ade6-M216 leu1 ura4-D18
JS18	h^{90} kan^{R}-Pnmt1-sme2-MS2loop-2 nat^{R}-CFP-mmi1 ade6-M216 leu1 ura4-D18
JS19	h^{90} kan^{R}-Pnmt1-sme2-5′-MS2loop-2-Tnmt nat^{R}-CFP-mmi1 ade6-M216 leu1 ura4-D18
JS20	h^{90} kan^{R}-Pnmt1-MS2loop-2-sme2-3′ nat^{R}-CFP-mmi1 ade6-M216 leu1 ura4-D18
JS21	h^{90} mei2::ura4^{+} nat^{R}-CFP-mmi1 ade6-M216 leu1 ura4-D18
JS22	h^{90} mei2::ura4^{+} kan^{R}-Pnmt1-sme2-MS2loop-2 nat^{R}-CFP-mmi1 ade6-M216 leu1 ura4-D18
JS23	h^{90} mei2::ura4^{+} kan^{R}-Pnmt1-sme2-5′-MS2loop-2-Tnmt nat^{R}-CFP-mmi1 ade6-M216 leu1 ura4-D18
JS24	h^{90} mei2::ura4^{+} kan^{R}-Pnmt1-MS2loop-2-sme2-3′ nat^{R}-CFP-mmi1 ade6-M216 leu1 ura4-D18
JS31	h^{90} CO2::Pnmt1-MS2loop-2-ura4-nat^{R} ade6-M216 leu1 ura4-D18
JS32	h^{90} CO2::Pnmt1-MS2loop-2-ssm4-nat^{R} ade6-M216 leu1 ura4-D18
JS33	h^{90} sme2-MS2loop-2 ade6-M216 leu1 ura4-D18

JS34	*h*^{90} *kan*^{R}*-Pnmt1-sme2-MS2loop-2 ade6-M216 leu1 ura4-D18*
JS35	*h*^{90} *mmi1-ts6-kan*^{R} *bsd*^{R}*-Pnmt1-sme2-MS2loop-2 ade6-M216 leu1 ura4-D18*
JS36	*h*^{90} *mei4::ura4*$^{+}$ *bsd*^{R}*-sme2-MS2loop-2 ade6-M216 leu1 ura4-D18*
JS37	*h*^{90} *mei4::ura4*$^{+}$ *mmi1::kan*^{R} *bsd*^{R}*-sme2-MS2loop-2 ade6-M216 leu1 ura4-D18*
JS38	*h*^{90} *bsd*^{R}*-sme2-MS2loop-2 ade6-M216 leu1 ura4-D18*
JS39	*h*^{90} *bsd*^{R}*-sme2-5'-MS2loop-2-Tnmt ade6-M216 leu1 ura4-D18*
JS40	*h*^{90} *bsd*^{R}*-sme2-3'-MS2loop-2 ade6-M216 leu1 ura4-D18*
JS41	*h*^{90}*/h*^{90} *bsd*^{R}*-sme2-MS2loop-2/bsd*^{R}*-sme2-MS2loop-2 ade6-M216/ade6-M210 leu1/leu1 ura4-D18/ura4-D18*
JS42	*h*^{90}*/h*^{90} *bsd*^{R}*-sme2-5'-MS2loop-2-Tnmt/bsd*^{R}*-sme2-5'-MS2loop-2-Tnmt ade6-M216/ade6-M210 leu1/leu1 ura4-D18/ura4-D18*
JS43	*h*^{90}*/h*^{90} *bsd*^{R}*-sme2-3'-MS2loop-2/bsd*^{R}*-sme2-3'-MS2loop-2 ade6-M216/ade6-M210 leu1/leu1 ura4-D18/ura4-D18*
JS44	*h*^{90}*/h*^{90} *bsd*^{R}*-sme2-DSRless-MS2loop-2/bsd*^{R}*-sme2-DSRless-MS2loop-2 ade6-M216/ade6-M210 leu1/leu1 ura4-D18/ura4-D18*
JS45	*h*$^{+}$*/h*$^{-}$ *mei4::ura4*$^{+}$*/mei4::ura4*$^{+}$ *mmi1::kan*^{R}*/mmi1::kan*^{R} *ade6-M216/ade6-M210 leu1/leu1 ura4-D18/ura4-D18*
JS46	*h*^{90} *pab2::hph*^{R} *bsd*^{R}*-sme2-MS2loop-2 ade6-M216 leu1 ura4-D18*
JS47	*h*^{90} *pla1-41-kan*^{R} *bsd*^{R}*-sme2-MS2loop-2 ade6-M216 leu1 ura4-D18*
JS48	*h*^{90} *clr4::kan*^{R} *bsd*^{R}*-sme2-MS2loop-2 ade6-M216 leu1 ura4-D18*
JS50	*h*^{90} *CO2::Pnmt1-MS2loop-2-ura4-nat*^{R} *kan*^{R}*-CFP-mmi1 ade6-M216 leu1 ura4-D18*
JS51	*h*^{90} *CO2::Pnmt1-MS2loop-2-ssm4-nat*^{R} *kan*^{R}*-CFP-mmi1 ade6-M216 leu1 ura4-D18*
JS52	*h*^{90} *mei4-ura4*$^{+}$*-kan*^{R}*-lacOx32 arg1::Padh41-4mCherry-lacI-NLS-hph*^{R} *LEU2-CFP-mmi1 ade6-M210 leu1 ura4*
JT926	*h*^{90} *sme2::ura4*$^{+}$ *ade6-M216 leu1 ura4-D18*
JV579	*h*^{90} *mmi1-ts3-kan*^{R} *ade6-M216 leu1*
JV582	*h*^{90} *mmi1-ts6-kan*^{R} *ade6-M216 leu1*
JY362	*h*$^{-}$*/h*$^{+}$ *ade6-M216/ade6-M210 leu1/leu1*
JY450	*h*^{90} *ade6-M216 leu1*
JZ807	*h*$^{-}$*/h*$^{+}$ *mei4::ura4*$^{+}$*/mei4::ura4*$^{+}$ *ade6-M216/ade6-M210 leu1/leu1 ura4-D18/ura4-D18*

Table S2. Oligonucleotides used in this study

For *lacO* integration

Name	Sequence
sme2-lacO F1	GTTGCTCCTATAGAAGAACA
sme2-lacO F2	TTAATTAACCCGGGGATCCGTTCGCGCAGATAAGCGCAAG
sme2-lacO R2	GTTTAAACGAGCTCGAATTCGATTAAATAGAATATTCGTACATG
sme2-lacO R1	CACCAAGTTTCTTAAAAGCG
mei4-lacO F1	ATGAGTCAAGTGGCTACTCAAGTGC
mei4-lacO F2	TTAATTAACCCGGGGATCCGTTGTTTCGTCAACATATATTATTAC
mei4-lacO R2	GTTTAAACGAGCTCGAATTCTTCTAAATAAAGTCATAGGAATGTA
mei4-lacO R1	GCGAGATAGTTAGAAGCGGAAGTGG

For quantitative PCR

Name	Sequence
act1-F	TGAGGAGCACCCTTGCTTGT
act1-R	TCTTCTCACGGTTGGATTTGG
mei4-F	AATGCGAAACTGAAGCATTG
mei4-R	TAGGATCGCCAAACCGATTA
sme2-5′-F	AAGACGGAATATGCATGCAAGA
sme2-5′-R	AACAAACCACAACACAAAGAAAGAGA
sme2-3′-F	GAAAATAAACAATAACCACAGCAAGCT
sme2-3′-R	ACAGCACAACCGAAGACCAAT
